# Desquamation in Kawasaki Disease

**DOI:** 10.3390/children8050317

**Published:** 2021-04-21

**Authors:** Ling-Sai Chang, Ken-Pen Weng, Jia-Huei Yan, Wan-Shan Lo, Mindy Ming-Huey Guo, Ying-Hsien Huang, Ho-Chang Kuo

**Affiliations:** 1 Department of Pediatrics, Kaohsiung Chang Gung Memorial Hospital and Chang Gung University College of Medicine, No.123 Da-Pei Road, Niaosong District, Kaohsiung 83301, Taiwan; joycejohnsyoko@gmail.com (L.-S.C.); chestnut761@gmail.com (J.-H.Y.); ilypinky@cgmh.org.tw (W.-S.L.); mindymhguo@yahoo.com.tw (M.M.-H.G.); yhhuang123@yahoo.com.tw (Y.-H.H.); 2 Kawasaki Disease Center, Kaohsiung Chang Gung Memorial Hospital, Kaohsiung 83301, Taiwan; 3 Congenital Structural Heart Disease Center, Department of Pediatrics, Kaohsiung Veterans General Hospital, Kaohsiung 813, Taiwan; kenpenweng@yahoo.com.tw; 4 Faculty of Medicine, School of Medicine, National Yang Ming University, Taipei 711, Taiwan; 5 Department of Physical Therapy, Shu-Zen Junior College of Medicine and Management, Kaohsiung 821, Taiwan

**Keywords:** desquamation, coronary artery lesion, Kawasaki disease

## Abstract

(1) Background: Desquamation is a common characteristic of Kawasaki disease (KD). In this study, we analyzed patients’ varying desquamation levels in their hands or feet, in correlation with clinical presentation, to assess the relationship. (2) Methods: We retrospectively reviewed children with KD. We analyzed their age, laboratory data before intravenous immunoglobulin (IVIG) treatment and coronary artery abnormalities (CAA) based on the desquamation level of their hands and feet. We classified the desquamation level from 0 to 3 and defined high-grade desquamation as grade 2 and 3. (3) Results: We enrolled a total 112 patients in the study. We found the hands’ high-grade desquamation was positively associated with age and segmented neutrophil percentage (*p* = 0.047 and 0.029, respectively) but negatively associated with lymphocyte and monocyte percentage (*p* = 0.03 and 0.006, respectively). Meanwhile, the feet’s high-grade desquamation was positively associated with total white blood cell counts (*p* = 0.033). Furthermore, we found that high-grade hand desquamation had less probability of CAA formation compared with that of a low grade (7.1% vs. 40.8%, *p* = 0.016). (4) Conclusions: This report is the first to demonstrate that the desquamation level of hands or feet in KD is associated with different coronary artery abnormalities and laboratory findings.

## 1. Introduction

Kawasaki disease (KD) is a form of acute febrile vasculitis that primarily affects children under the age of 5 years old, particularly those of Asian descent. KD could cause coronary artery dilatation or aneurysm formation if treatment is not received in a timely manner. KD is characterized by prolonged fever of more than 5 days, as well as four of the following five typical features: bilateral non-exudative conjunctivitis, oral changes (erythema in lip or oropharyngeal mucosa, strawberry tongue, fissure lip), cervical lymphadenopathy, skin rash and changes in extremities [1]. While KD has been suggested to have an infectious association and gene susceptibility, no consistent causative pathogen was identified and the etiology is still not well understood. The affected coronary arteries undergo an inflammatory process with neutrophilic infiltration, upregulation of cytotoxic T cells and myofibroblastic proliferation, which pushes coronary arteries to stenosis [2,3]. While some coronary changes may resolve within 1 to 2 years [1], pediatricians are concerned some may remain life-long sequalae.

The desquamation of hands and feet, which may extend to palms and soles, is a subacute phase of extremity change and usually occurs 2 to 3 weeks after fever onset [1]. According to a number of studies, the prevalence of periungual peeling ranges from 68% to 98% [4,5,6]. The pathogenesis of desquamation in KD is not clear, but previous studies have shown the association between skin peeling with clinical signs and laboratory findings [6,7]. Nevertheless, researchers have not compared the clinical presentation with the severity of desquamation in KD. In this study, we aimed to assess the level of hand and foot desquamation, from fingertips (toes) to palm (sole) and compare them with their clinical characteristics in case studies.

## 2. Materials and Methods

We retrospectively reviewed children with KD who had follow-up visits in Kaohsiung Chang Gung Memorial Hospital’s pediatric outpatient department during 2018 to 2019. Patients who were treated in another hospital during the acute phase or cases with incomplete medical records were excluded. KD was diagnosed in accordance with the American Heart Association (AHA) criteria, which includes fever for more than 5 days and typically having four of five clinical features (bilateral non-exudative conjunctivitis, oral changes, cervical lymphadenopathy (>1.5 cm in diameter), hand and feet edema or desquamation and polymorphism skin rash), while incomplete KD was diagnosed as prolonged fever and two or three clinical features, plus C-reactive protein (CRP) ≥ 30 mg/L and/or ESR ≥ 40 mm/hour and supplemental laboratory data (anemia by age, platelet count ≥/mm^3^ after 7th day of fever, albumin ≤ 3 g/dL, elevated ALT level, white blood cell (WBC) count ≥ /mm^3^, urine ≥ 10 WBC/high power field) or echocardiography findings. Coronary artery abnormalities (CAA) are defined as: (1) left anterior descending (LAD) coronary artery or right coronary artery (RCA) Z score of ≥2.5; (2) coronary artery aneurysm; (3) the presence of ≥3 other suggestive features, including decreased left ventricular function, mitral regurgitation, pericardial effusion, Z scores in LAD coronary artery or RCA of 2 to 2.5 [1]. IVIG resistance was defined as persistent fever at least 48 h after initial IVIG therapy where a second IVIG infusion had to be administered to patients. 

**Figure 1:**
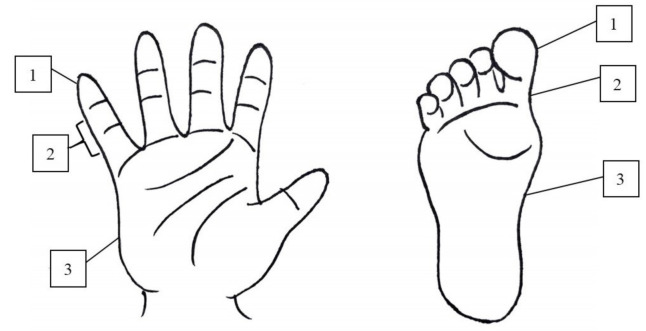
The illustration to identify the grade of desquamation.

**Figure 2:**
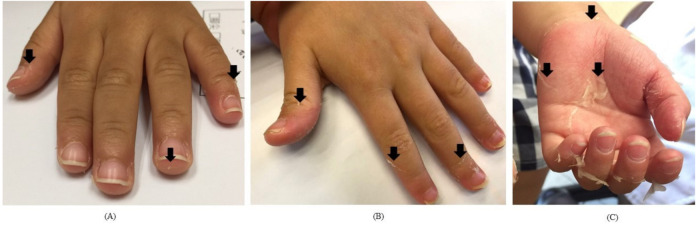
The desquamation of hands; the black arrows point out the margin of peeling; (**A**): grade 1; (**B**): grade 2; (**C**): grade 3.

**Figure 3:**
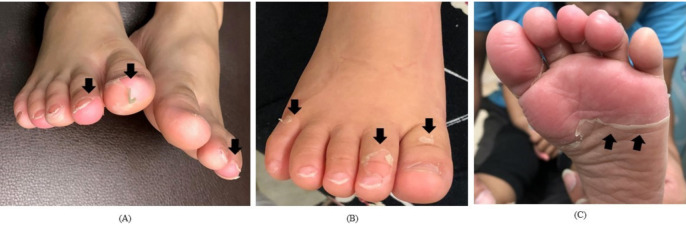
The desquamation of feet; the black arrows point out the margin of peeling; (**A**): grade 1; (**B**): grade 2; (**C**): grade 3.

We classified groups into desquamation positive or not and then further divided into “hand desquamation” and “foot desquamation”. We provided an illustration (Figure 1) for caregivers to identify the desquamation grade because there was no standardized assessment of skin peeling in KD during the period of study. In hands and feet, a classified number of 0 meant no desquamation, while 1 to 3 represented fingertip (toe tip), between fingertip (toe tip) and palm (sole) (Figure 2 and Figure 3). High-grade desquamation was defined as levels 2 and 3, while low-grade referred to level 1 (limited to fingertips or toe tips). Patients were divided into groups according to “desquamation or not” and “desquamation grades”. We analyzed patients’ age and laboratory data within 3 days prior to the administration of intravenous immunoglobulin (IVIG) and follow-up echocardiography series from admission to 1 to 2 years after disease onset. This study was approved by the Institutional Review Board of Chang Gung Memorial Hospital (201600714A3).

We performed the Kolmogorov-Smirnova test to examine data distribution, the Kruskal-Wallis and Mann-Whitney tests for continuous variable analysis and the Chi-square test for categorical variables. To determine the cut-off value, we used the receiver operating characteristic (ROC) curve, Youden index and minimal distance. Data are presented as percentage and median with interquartile range (IQR). Results were considered statically significant when *p*-values < 0.05. All statistical analysis was performed using SPSS 22.0 (SPSS, Inc., Chicago, IL, USA).

## 3. Results

This study included a total of 112 patients and 92 of them (82.1%) had hand or foot desquamation. The laboratory data and IVIG resistant rate demonstrated no significant difference between patients with and those without desquamation (Table 1). We further analyzed data based on “hand desquamation” and “foot desquamation”. In both categories, we further classified into sub-groups according to “Desquamation positive or negative” and “High-grade or low-grade” (Table 2 and Table 3).

In the “hand desquamation” group (Table 2), 91 patients were positive for hand desquamation and the distribution of grades 1, 2 and 3 were 77, 7 and 7 cases, respectively. The median age of the hands’ desquamation positive group was older than that of negative group (*p* = 0.046). Furthermore, older age (*p* = 0.047), elevated segmented WBC percentage (*p* = 0.029), lower lymphocyte (*p* = 0.03) and lower monocyte (*p* = 0.006) percentage were all associated with high-grade desquamation. Regarding age, the area under the curve (AUC) of hand desquamation positive is 0.64, with an optimal cut-off value of 2 years old. The desquamation positive rate was 97% (≧2 years old) and 74.7% (<2 years old), respectively (*p* = 0.006). 

In the “foot desquamation” group (Table 3), 63 patients were positive for foot desquamation and the distribution of grades 1, 2 and 3 were 54, 2 and 7 cases, respectively. The desquamation positive group had higher CRP but lower aspartate transaminase (AST) levels (*p* = 0.023 and 0.045, respectively), while the high-grade desquamation group had a higher WBC count (*p* = 0.033). We observed no significant difference in the IVIG-resistant rate between hand and foot desquamation. There is a significant correlation between the degree of peeling of the hands and that of the feet (high grade vs. low grade, negative vs. positive peeling between hands and feet, all *p* < 0.001).

With regard to coronary artery abnormalities (CAA), the total desquamation and hand and foot desquamation incidences were similar in patients with CAA and those without (Table 4). We further analyzed the subgroups and found that patients with CAA formation had a significantly lower incidence of only 3% accompanied with high-grade hand desquamation (*p* = 0.016). Furthermore, high-grade hand peeling had a decreased incidence of CAA formation than low-grade hand peeling (7.1% vs. 40.8%, *p* = 0.016) in two-year series echocardiography, while we had no significant finding in the feet subgroups.

## 4. Discussion

To the best of our knowledge, this study is the first to discuss different levels of desquamation associated with the clinical symptoms and signs of KD. Epidermal turnover and desquamation are natural processes in mammals [8], but some diseases may progress these processes. For example, the toxin secreted by Staphylococcus aureus is related to the cleavage of keratinocyte junction and cell-to-cell adhesion in the epidermis [9], which may cause Staphylococcus aureus scalded skin syndrome. The activation of cutaneous T cells induced by the superantigen of streptococcus results in upregulating skin-homing capacity and the rash of scarlet fever is followed by peeling in response to cytokine release and activated T-cell infiltration [10]. Hand-foot-mouth disease caused by the enterovirus also contributes to skin desquamation, but the related pathophysiology remains poorly understood [11,12]. Although the desquamation of KD presents as part of immune reaction, the exact mechanism is still not well known. In animal experiments, bacilli Calmette Guérin (BCG) injection and heat shock protein (HSP) administration resulted in coronary artery inflammation and tail peeling in programmed death-1 gene knockout mice [13]. HSP was speculated as a triggering factor of KD. BCG hyperresponsiveness due to the T-cell-mediated delayed reaction and cross-reactivity between mycobacterial HSP and human homologue HSP was observed as the characteristic feature in KD patients [14,15]. In addition, to BCG and HSP in mouse research models, the peeling phenomenon of KD has also been reported to be related to the secretion of interleukin (IL)-2 responsible for the lymphocytes infiltrating damaged tissues [16].

Hand and foot desquamation have distinct characteristics in this study. The desquamation in KD mostly occurred during the convalescent phase of the disease [17]. We did not observe any significant difference in the laboratory findings between patients with and those without desquamation. However, we further classified patients into hand or foot desquamation and began to observe differences. Hand desquamation was associated with older age and high-grade hand peeling was related to older age, elevated segmented WBC percentage, lower lymphocyte and monocyte percentages. Meanwhile, foot desquamation was associated with elevated CRP and AST and high-grade foot desquamation was related to increased WBC count. In the AHA diagnosis criteria of atypical KD, supplemental laboratory tests include WBC count, CRP and ALT level, all of which indicate the inflammation status of KD. Wang et al. revealed desquamation positive KD patients had lower WBC counts, higher band-form percentage and elevated ALT level, but the age, gender, percentage neutrophils, hemoglobulin, CRP and platelet count were similar [6]. In another study analyzing desquamation in KD, Kim et al. revealed that patients with desquamation had higher platelet counts and elevated AST and ALT [7]. In our study, the WBC counts were only associated with high-level foot desquamation; in contrast, higher AST levels were negatively related to foot desquamation. Both the band-form percentage and ALT level were similar in our subgroups. The two aforementioned studies did not further discuss differences in laboratory findings in peeling severity in either the hands or the feet.

Interestingly, we found that high-grade hand peeling was related to a decreased incidence of CAA formation. Kim et al. showed no difference between groups with or without desquamation regarding coronary artery lesions [7]. However, Wang et al. also indicated that cases who did not peel had a higher probability of aneurysm formation [6] and another study revealed that recurrent skin peeling in KD was significantly less frequent in patients with coronary dilatation or aneurysm formation [18]. Desquamation seems to have a protective effect on CAA formation. Although the mechanism is not clear, we suppose that the more cytokines released to facilitate skin peeling, the fewer inflammatory factors in coronary arteries. In another study of cutaneous microcirculatory status in patients with Kawasaki disease, under the dynamic capillaroscopy, the capillary blood cell velocity decreased because vessel dilatation and correlated with increased coronary artery diameter in afebrile phase [19]. In contrast, we hypothesize the vessels not dilated with increased velocity and inflammatory substance carried out by rich blood supply enhanced the turnover of epidermal. Further research is required to identify the pathophysiology. It is not known whether the difference between the formation mechanism of low and high exfoliation is related to the role of type 2 inflammation plays in CAA. The absolute counts of low-affinity immunoglobulin E receptor CD23+ macrophages/monocytes in KD patients with CAA were lower than those in patients without CAA [20]. Post-IVIG eosinophilia was associated with the lower rate of CAA [21]. At the same time, Hwang et al. performed the study which indicated that KD children without CAA were more likely to develop allergic diseases [22]. For example, in atopic dermatitis, peeling is an inflammatory reaction.

This study had certain limitations. We conducted a retrospective review of medical records and some cases of desquamation were identified based on the reporting of family history, both of which may have recall bias and variable accuracy. The day number from the onset may be a significant factor. There is a possibility that the later a patient is diagnosed, the stronger inflammations on the hands and feet. Furthermore, this study was a single center experience, which is not sufficient for reflecting the general condition nationwide. As for CAA formation, some patients who were diagnosed as having KD under 2 years of age, a longer echocardiography is needed to observe coronary artery changes. Both multi-center studies and longer follow-up times are needed to analyze the various grades of desquamation in KD with clinical features.

## 5. Conclusions

This report is the first to study various severities of hand and foot periungual desquamation in KD associated with coronary artery abnormalities. Pediatricians should be aware of prolonged fever and periungual desquamation with unknown origin. Furthermore, the risk of coronary artery changes cannot be overlooked and we should be particularly cautious with those who have low-grade hand desquamation in KD. Further prospective studies are needed to confirm the preliminary findings.

## Figures and Tables

**Table 1:** Patients’ laboratory data—desquamation positive or negative.

	Desquamation Negative	Desquamation Positive	*p* Value
**Total**	20 (17.9%)	92 (82.1%)	
**Male gender**	14/20 (70.0%)	52/92 (56.5%)	0.322
**Age**	1.1 (0.6–1.6)	1.4 (0.8–2.6)	0.063
**WBC (1000/mm^3^)**	12.7 (9.7–16.4)	12.5 (10.1–14.4)	0.536
**Hemoglobulin (g/dL)**	11.0 (10.3–11.9)	11.1 (10.3–12.0)	0.811
**Platelet (1000/mm^3^)**	333.0 (253.5–434.8)	361.0 (285.5–435.8)	0.416
**Segmented WBC (%)**	53.7 (45.0–65.6)	61.0 (47.1–72.0)	0.173
**Band WBC (%)**	0 (0–0)	0 (0–0)	1.000
**Lymphocyte (%)**	29.2 (25.4–45.1)	29.2 (20.0–40.0)	0.368
**Monocyte (%)**	7.1 (3.2–10.1)	5.0 (3.8–8.0)	0.277
**CRP (mg/L)**	35.8 (14.7–64.0)	58.4 (28.6–116.1)	0.078
**AST (IU/L)**	42.0 (32.5–62.8)	35.0 (25.0–75.0)	0.342
**ALT (IU/L)**	23.5 (16.0–93.3)	45.0 (18.0–92.8)	0.268
**Albumin (g/dL)**	4.0 (3.5–4.3)	3.9 (3.6–4.2)	0.852
**Urine WBC (/μL)**	3.0 (0–34.0)	6.0 (0–24.0)	0.950
**IVIG resistant rate**	1/20 (5.0%)	6/92 (6.5%)	1.000

ALT, alanine aminotransferase; AST, aspartate aminotransferase; CRP, C-reactive protein; IVIG, intravenous immunoglobulin; WBC, white blood cells.

**Table 2:** Patients’ laboratory data of Kawasaki disease before IVIG (with hands’ desquamation positive or not).

	Hands’ Desquamation Negative	Hands’ DesquaMation Positive	*p* Value	Low-Grade Desquamation	High-Grade Desquamation	*p* Value
**Total**	21	91		77	14	
**Male gender**	14/21 (66.7%)	52/91 (57.1%)	0.470	44/77 (57.1%)	8/14 (57.1%)	1.000
**Age**	1.0 (0.6–1.6)	1.5 (0.7–2.6)	0.046 *	1.4 (0.7–2.5)	2.2 (1.3–4.2)	0.047 *
**WBC (1000/mm^3^)**	12.4 (9.8–16.2)	12.5 (10.1–14.4)	0.568	12.5 (9.8–14.4)	12.4 (11.4–14.8)	0.460
**Hemoglobulin (g/dL)**	10.7 (10.3–11.9)	11.1 (10.3–12)	0.723	11.1 (10.2–12)	11.2 (10.3–11.8)	0.697
**Platelet (1000/mm^3^)**	341.0 (254.0–433.5)	360.0 (284.0–437.0)	0.519	352.0 (287.0–431.0)	365.5 (262.3–447.0)	0.980
**Segmented WBC (%)**	51.3 (45.3–65.3)	61.0 (48.1–72.0)	0.123	59.0 (46.2–70.7)	69.8 (60.7–76.8)	0.029 *
**Band WBC (%)**	0 (0–0)	0 (0–0)	0.900	0 (0–0)	0 (0–1)	0.702
**Lymphocyte (%)**	29.7 (25.6–45.7)	29.1 (20.0–40.0)	0.236	30.0 (21.6–40.1)	20.1 (14.6–31.1)	0.030 *
**Monocyte (%)**	7.0 (3.1–9.7)	5.0 (3.9–8.0)	0.412	5.5 (4.0–8.9)	4.1 (2.8–4.9)	0.006 *
**CRP (mg/L)**	37.4 (15.3–65.0)	58.2 (28.1–115.1)	0.145	57.7 (29.0–104.5)	101.3 (22.2–177.3)	0.324
**AST (IU/L)**	42.0 (34.0–86.0)	35.0 (25.0–69.8)	0.214	35.0 (26.0–64.5)	34.0 (25.0–178.5)	0.923
**ALT (IU/L)**	24.0 (16.0–95.0)	43.0 (18.0–88.0)	0.395	39.0 (18.3–82.5)	55.0 (15.0–237.0)	0.935
**Albumin (g/dL)**	4.0 (3.5–4.3)	3.9 (3.6–4.2)	0.852	3.9 (3.5–4.2)	4.1 (3.9–4.4)	0.070
**Urine WBC (/μL)**	3.0 (0–28.5)	6.0 (0–24.0)	0.817	6.0 (0–21.0)	6.0 (0–145.5)	0.395
**IVIG resistant rate**	1/21 (4.8%)	6/91 (6.6%)	1.000	5/77(6.5%)	1/14 (7.1%)	1.000

ALT, alanine aminotransferase; AST, aspartate aminotransferase; CRP, C-reactive protein; IVIG, intravenous immunoglobulin; WBC, white blood cells. * *p* < 0.05.

**Table 3:** Patients’ laboratory data of Kawasaki disease before IVIG (with feet’s desquamation positive or not).

	Feet’s Desquamation Negative	Feet’s Desquamation Positive	*p* Value	Low-Grade Desquamation	High-Grade Desquamation	*p* Value
**Total**	39	63		54	9	
**Male gender**	27/39 (69.2%)	32/63 (50.8%)	0.067	27/54 (50.0%)	5/9 (55.6%)	1.000
**Age**	1.1 (0.7–1.9)	1.6 (0.7–2.5)	0.185	1.6 (0.7–2.2)	1.4 (1.1–3.6)	0.327
**WBC (1000/mm^3^)**	11.3 (9.2–15.2)	12.5 (10.5–14.4)	0.610	12.3 (10.2–14.0)	14.0 (12.8–17.0)	0.033 *
**Hemoglobulin (g/dL)**	11.3 (10.3–12.0)	10.9 (10.2–11.7)	0.484	10.9 (10.2–11.9)	10.7 (10.2–11.7)	0.746
**Platelet (1000/mm^3^)**	325.0 (255.0–431.0)	375.0 (299.0–441.0)	0.094	376.5 (297.3–442.3)	371.0 (313.0–449.5)	0.912
**Segmented WBC (%)**	56.2 (44.6–68.0)	62.0 (51.0–72.0)	0.300	61.0 (49.8–69.8)	63.0 (58.4–74.3)	0.249
**Band WBC (%)**	0 (0–0)	0 (0–0)	0.961	0 (0–0)	0 (0–0)	0.528
**Lymphocyte (%)**	29.7 (20.0–44.3)	29.0 (21.2–39.0)	0.498	28.5 (21.8–39.3)	29 (16.9–33.3)	0.539
**Monocyte (%)**	7.0 (3.8–10.0)	4.7 (3.1–7.0)	0.070	5.0 (3.4–7.9)	4.0 (3.0–4.8)	0.098
**CRP (mg/L)**	39.3 (14.1–67.6)	58.3 (32.1–122.1)	0.023 *	58.3 (32.1–119.8)	76.3 (29.3–179.7)	0.845
**AST (IU/L)**	42.5 (32.3–93.5)	34.0 (25.0–68.5)	0.045 *	34.0 (25.5–99.5)	25.0 (21.0–47.0)	0.155
**ALT (IU/L)**	34.5 (18.0–107.3)	38.5 (17.0–74.3)	0.635	47.5 (17.8–77.3)	23.5 (14.0–71.0)	0.495
**Albumin (g/dL)**	3.9 (3.5–4.3)	4.0 (3.6–4.2)	0.905	4.0 (3.6–4.2)	4.0 (3.6–4.4)	0.469
**Urine WBC (/μL)**	3.0 (0–34.0)	6.0 (0–24.3)	0.763	6.0 (0–21.8)	4.5 (0–142.8)	0.931
**IVIG resistant rate**	2/39 (5.1%)	4/63 (6.3%)	1.000	4/54	0/9	1.000

ALT, alanine aminotransferase; AST, aspartate aminotransferase; CRP, C-reactive protein; IVIG, intravenous immunoglobulin; WBC, white blood cells. * *p* < 0.05.

**Table 4:** CAA formation incidence in subgroups.

	CAA ^a^ (−)	CAA (+)	*p* Value
Total desquamation (+)	59/71 (83.1%)	33/41 (80.5%)	0.800
Hands			
Desquamation (+)	58/71 (81.7%)	33/41 (80.5%)	1.000
High-grade	13/58 (22.4%)	1/33 (3.0%)	0.016 *
Feet			
Desquamation (+)	42/67 (62.7%)	21/35 (60.0%)	0.832
High-grade	8/42 (19.0%)	1/21 (4.5%)	0.159

^a^ CAA, Coronary artery abnormalities. * *p* < 0.05.

## Data Availability

The datasets generated and analyzed during the current study are not publicly available due to strict ethical regulation of information privacy, but are available from the corresponding author Dr. Ho-Chang Kuo on reasonable request.

## Abbreviations

ALT: Alanine Aminotransferase; AHA: American Heart Association; AST: Aspartate Aminotransferase; AUC: area under the curve; CAA: Coronary artery abnormalities; CRP: C-reactive protein; ESR: Erythrocyte sedimentation rate; HFMD: Hand, foot and mouth disease; IQR: interquartile range; IVIG: intravenous immunoglobulin; KD: Kawasaki disease; LAD: left anterior descending artery; RCA: right coronary artery; ROC curve: receiver operating characteristic curve; SSSS: Staphylococcal scalded skin syndrome; WBC: White blood cells.
